# Bioactivity Guided Study for the Isolation and Identification of Antidiabetic Compounds from Edible Seaweed—*Ulva reticulata*

**DOI:** 10.3390/molecules27248827

**Published:** 2022-12-12

**Authors:** Pullikaparambil Sasidharan Unnikrishnan, Andhere Animish, Gunabalan Madhumitha, Krishnamurthy Suthindhiran, Mangalam Achuthananthan Jayasri

**Affiliations:** 1 Marine Biotechnology and Bioproducts Laboratory, Vellore Institute of Technology, School of Biosciences and Technology, Vellore 632014, India; 2 Chemistry of Heterocycles and Natural Products Research Laboratory, Vellore Institute of Technology, School of Advanced Sciences, Vellore 632014, India

**Keywords:** *Ulva reticulata*, diabetes, α-amylase, α-glucosidase, bioactive compounds

## Abstract

Managing diabetes is challenging due to the complex physiology of the disease and the numerous complications associated with it. As part of the ongoing search for antidiabetic chemicals, marine algae have been demonstrated to be an excellent source due to their medicinal properties. In this study, *Ulva reticulata* extracts were investigated for their anti-diabetic effect by examining its inhibitory effects on α-amylase, α-glucosidase, and DPP-IV and antioxidant (DPPH) potential in vitro and its purified fraction using animal models. Among the various solvents used, the Methanolic extract of *Ulva reticulata* (MEUR) displayed the highest antidiabetic activity in both in vitro and in vivo; it showed no cytotoxicity and hence was subjected to bioassay-guided chromatographic separation. Among the seven isolated fractions (F1 to F7), the F4 (chloroform) fraction exhibited substantial total phenolic content (65.19 μg mL^−1^) and total flavonoid content (20.33 μg mL^−1^), which showed the promising inhibition against α-amylase (71.67%) and α-glucosidase (38.01%). Active fraction (F4) was further purified using column chromatography, subjected to thin-layer chromatography (TLC), and characterized by spectroscopy techniques. Upon structural elucidation, five distinct compounds, namely, Nonane, Hexadecanoic acid, 1-dodecanol, Cyclodecane methyl, and phenol, phenol, 3,5-bis(1,1-dimethylethyl) were identified. The antidiabetic mechanism of active fraction (F4) was further investigated using various in vitro and in vivo models. The results displayed that in in vitro both 1 and 24 h in vitro cultures, the active fraction (F4) at a concentration of 100 μg mL^−1^ demonstrated maximum glucose-induced insulin secretion at 4 mM (0.357 and 0.582 μg mL^−1^) and 20 mM (0.848 and 1.032 μg mL^−1^). The active fraction (F4) reduces blood glucose levels in normoglycaemic animals and produces effects similar to that of standard acarbose. Active fraction (F4) also demonstrated outstanding hypoglycaemic activity in hyperglycemic animals at a dose of 10 mg/kg B.wt. In the STZ-induced diabetic rat model, the active fraction (F4) showed a (61%) reduction in blood glucose level when compared to the standard drug glibenclamide (68%). The results indicate that the marine algae *Ulva reticulata* is a promising candidate for managing diabetes by inhibiting carbohydrate metabolizing enzymes and promoting insulin secretion.

## 1. Introduction

Diabetes mellitus (DM) is the most common metabolic disorder affecting various body organs and is associated with other complications such as coronary heart disease, stroke, liver damage, nephropathy, retinopathy, and peripheral neuropathy [1]. Since DM has become more prevalent in recent years, it has significantly impacted individual health [2]. The disease is characterized by hyperglycemia due to abnormalities in insulin production or action or a combined effect of both [3]. The reduction of postprandial hyperglycemia (PPHG) is the best therapeutic approach for early DM treatment and is associated with chronic vascular complications [4]. Currently, enzyme inhibitors of important carbohydrate digesting enzymes such as α-glucosidase and α-amylase in combination with other hypoglycemic medications or insulin, or alone, can manage PPHG, but it is associated with a variety of gastrointestinal adverse effects [5]. Carbohydrate absorption from the small intestine is inhibited by α-amylase and α-glucosidase inhibitors like acarbose, miglitol, and Voglibose, as they block enzymes that transform complex non-absorbable polysaccharides into simple absorbable carbohydrates by competing with them and delaying their absorption, thereby helping in managing postprandial hyperglycemia [6]. Another strategy is to reduce the action of the ubiquitous enzyme DPP-4 by using oral anti-diabetic medicines such DPP-4 inhibitors like sitagliptin, vildagliptin, which boost the activity of incretins from the intestine [7]. Delaying the degradation of incretin hormones enhances insulin secretion and glycogenesis by promoting α and β cell functions, consequently suppressing the PPHG [8]. Current synthetic drugs with this action, however, have unfavorable side effects, including abdominal distension, flatulence, meteorism, diarrhea, upper respiratory tract and urinary infections, renal dysfunction, metabolic acidosis, including diabetic ketoacidosis, cirrhosis, inflammatory bowel disease, ulcers of the intestine, partial intestinal obstruction, and digestive problems [9,10,11]. Another developing area in diabetes therapy is the use of sugar derivatives and iminosugars to precisely modify carbohydrate structures and regulate in vivo glycosidase activity [12,13]. Hence, there is considerable interest in searching for better and safer antidiabetic agents from natural resources, particularly seaweeds, which remain an active area for pharmaceutical research [14].

Various industrial and medical applications can be derived from marine algae [15]. Marine macroalgae or seaweeds are a rich source of bioactive compounds and have great potential in pharmaceutical, functional food, and biomedical sectors [16,17,18]. Several studies have reported seaweed’s bioactivities, including anti-inflammatory, antioxidant, antitumor, and antidiabetic activity [19,20,21,22,23,24,25]. Nevertheless, most of the studies on the bioactivities of seaweed are carried out in crude extracts, and thus characterization of active compounds is lacking. *Ulva* is one of the most widely distributed green algae and collected for consumption in worldwide due to its special valued nutrients with a long history. Specifically the species of *Ulva* are rich in bioactive compounds and the species *Ulva lactuca* reported for its hypoglycemic effect in aged diabetic models [26]. In this regard, the present study aims to isolate individual constituents from edible seaweed *Ulva reticulata* through bioassay-guided fractionation and to evaluate its respective antidiabetic activity using in vitro and in vivo models.

*Ulva reticulata* is edible green seaweed with a high content of minerals, vitamins, and other phytoconstituents. Its balanced amino acid profile makes this a potential dietary alternative [27,28]. This seaweed is widely distributed in Asian countries and consumed as fresh, dried ingredients in prepared foods [27,28,29,30]. As part of our ongoing research on seaweeds, this study describes the antidiabetic effects (α-amylase, α-glucosidase, and DPP-IV inhibition) of *Ulva reticulata* followed by bioactivity-guided isolation and characterization of compounds using chromatographic and spectroscopic techniques.

## 2. Results

### 2.1. In Vitro α-Amylase, α-Glucosidase, and DPP-IV Inhibition Study

**Figure 1:**
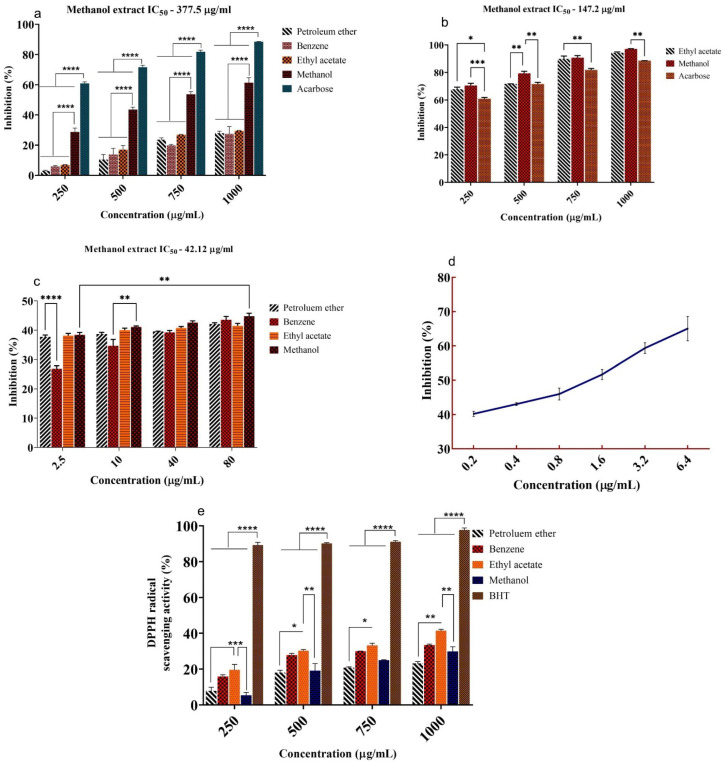
In vitro antidiabetic (α-amylase, α-glucosidase, and DPP-IV) and antioxidant activity of *U. reticulata*. (**a**) α-amylase, (**b**) α-glucosidase, (**c**) DPP-IV inhibitory, (**d**) Diprotin A and (**e**) antioxidant activity of seaweed extracts. Absorbance was measured at 540, 405, and 517 nm, respectively. All the extracts were compared with standards acarbose, Diprotin A, and BHT, where *n* = 3 for each sample with S.E.M. The mean values were analyzed using two-way ANOVA (Tukey’s multiple comparisons test). **** p < 0.0001, *** *p* < 0.001, ** *p* < 0.01, * *p* < 0.05 considered as significant.

The inhibitory effect of *U. reticulata* extracts on α-amylase, α-glucosidase, and DPP-IV was investigated, and its respective IC_50_ values were given in Figure 1a–c. Among the five extracts, the methanolic extract of *U. reticulata* (MEUR) displayed significant inhibitory activity against α-amylase (61%) at 1000 μg/mL concentration, against α-glucosidase (97%) at 1000 μg/mL concentration and against DPP-IV (44%) at 80 μg/mL concentration. The IC_50_ values for MEUR against α-amylase (377.5 μg/mL), α-glucosidase (147.2 μg/mL), and DPP-IV (42.12 μg/mL) were compared with standard acarbose (171.8 μg/mL^−1^) and Diprotin A (2.45 μg/mL) (Figure 1d).

### 2.2. Free Radical Scavenging Activity (DPPH)

The free radical scavenging activity (DPPH) of *U. reticulata* was moderate. Among the various extracts studied, the ethyl acetate extract of *U. reticulata* (1000 μg/mL) showed the highest free radical scavenging activity (41%) against DPPH, whereas the standard BHT exhibited 97% (Figure 1e). Benzene (33%), methanol (30%), and petroleum ether (23%) extracts showed less scavenging activity when compared to ethyl acetate.

### 2.3. Measurement of Cell Viability and In Vitro Hemolytic Activity of MEUR

**Figure 2:**
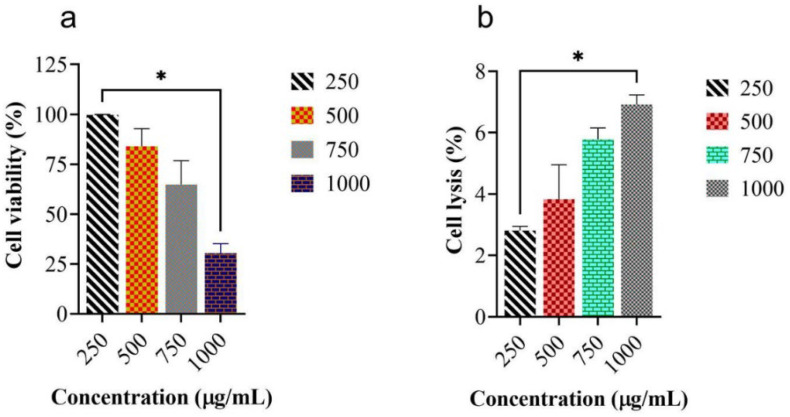
Measurement of cell viability (MTT assay) and hemolytic activity of *U. reticulata*. (**a**) Cell viability was determined by MTT assay. J774 cells treated with MEUR. Absorbance was measured at 630 nm. Values are mean ± S.D (*n* = 3). (**b**) The hemolytic activity of MEUR, Absorbance was measured at 630 nm. Values are means ± S.E.M. (*n* = 3). The mean values were analyzed using Dunn’s multiple comparison test. * *p* < 0.05 considered as significant.

The toxicological evaluation of MEUR was assessed by measuring the cell viability and hemolytic activity using mouse macrophage cells (J774) and human red blood cells, respectively. The results showed that a lower concentration of 250 μg/mL MEUR showed maximum viability (99%) for 24 h (Figure 2a). Similarly, MEUR did not show any hemolysis or erythrocyte membrane damage against human red blood cells at all the tested concentrations. There was only 6.92% cell lysis at 1000 μg/mL, and at a concentration of 250 μg/mL, MEUR showed 2.81% lysis against human red blood cells (Figure 2b).

### 2.4. Effect of MEUR on STZ-Induced Diabetic Rats

We examined the hypoglycaemic effect of MEUR (250 mg kg^−1^ B.wt.) on STZ-induced diabetic rats (Table 1). Fasting blood glucose (FBG) level was measured at regular intervals (0, 7, 14, 21 & 28 days) in normal and diabetic rats treated with MEUR and standard antidiabetic drug glibenclamide (0.25 mg kg^−1^ B.wt.). The FBG level of untreated rats (diabetic control) remained significantly increasing (˃329 mg/dL) throughout the study. Oral administration of MEUR (46.21%) and glibenclamide (73.05%) in treated rats showed a notable reduction in the blood glucose level compared to the untreated diabetic rats.

**Figure 3:**
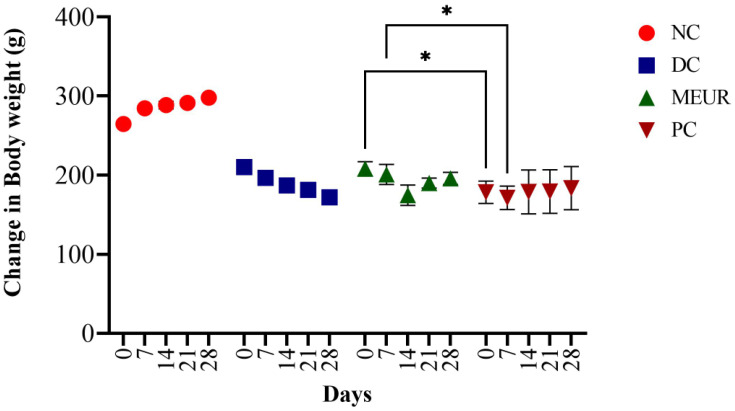
Effect of MEUR on body weight of STZ-induced diabetic rats. Each value is expressed as mean ± S.E.M. (*n* = 6). The mean values were analyzed using two-way ANOVA (Tukey’s multiple comparisons test). * *p* < 0.05 considered as significant.

**Figure 4:**
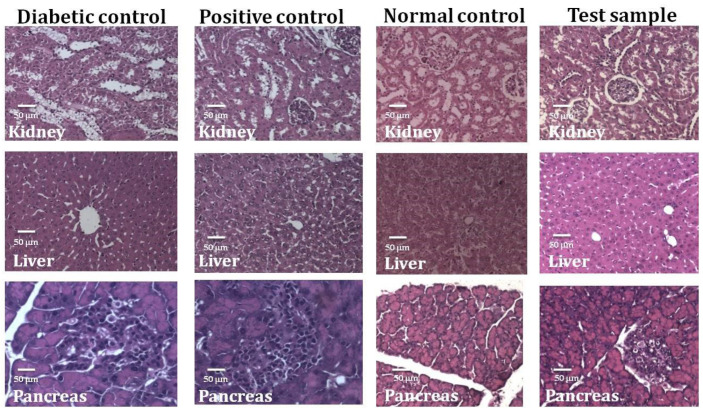
Histopathological changes in kidney, liver, and pancreas of STZ-induced diabetic rats. Photomicrograph (50 μM) of kidney, liver, and pancreas (hematoxylin & eosin) of Diabetic control, Positive control, Normal control, and MEUR.

The body weight of diabetic rats was reduced compared to normal rats; however, after administration of MEUR and glibenclamide for 28 days, their body weight increased compared to the untreated diabetic rats (Figure 3). There was also a change in serum parameters, including cholesterol, triglycerides, urea, and liver markers (ALT & AST) in STZ-induced diabetic rats (Table 2). After the administration of MEUR and glibenclamide, there was a significant reduction in the amount of cholesterol, triglycerides, ALT, and AST compared to untreated diabetic rats. Other parameters such as total protein, urea, albumin, and globulin were similar to normal control. Histopathology analysis reveals an extensive alteration in the morphology of the kidney, liver, and pancreas of diabetic rats (Figure 4). The pathological changes observed in diabetic rats were retained to normal after administering MEUR and glibenclamide for 28 days.

Among the 5 extracts, the methanolic extract of *Ulva reticulata* (MEUR), exhibiting the highest antidiabetic activity in both in vitro and in vivo studies, was subjected to chromatographic separation using a silica gel column (60–120 mesh).

### 2.5. In Vitro α-Amylase and α-Glucosidase Inhibitory Activity of Isolated Fractions from MEUR

**Figure 5:**
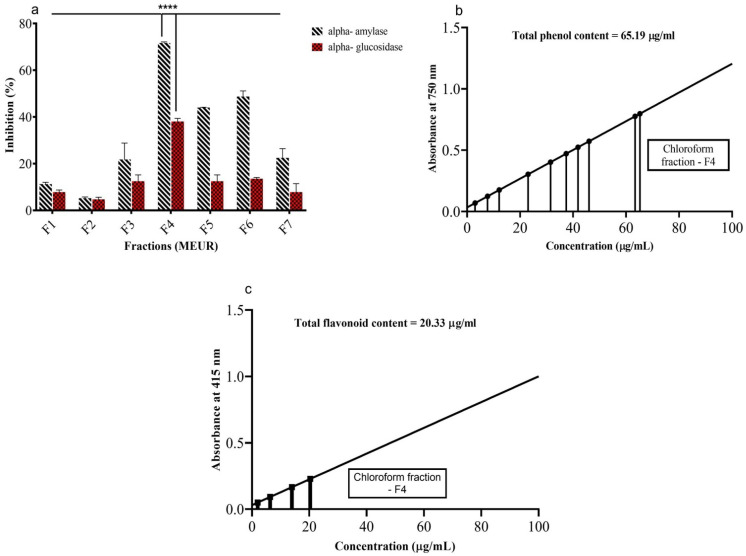
In vitro α-amylase, α-glucosidase inhibitory activity, and determination of total phenols and flavonoids of isolated fractions from MEUR. (**a**) In vitro α-amylase, α-glucosidase, (**b**) total phenols, and (**c**) flavonoids of various fractions of MEUR. Absorbance was measured at 540 nm (α-amylase) and 405 nm (α-glucosidase). Mean values were analyzed using two-way ANOVA (Tukey’s multiple comparisons test). **** *p* < 0.0001 considered as significant.

MEUR loaded onto silica gel column (60–120 mesh) were eluted with various solvents, starting with less polarity solvent in the sequence, such as hexane (F1), benzene (F2), Dichloromethane (F3), chloroform (F4), ethyl acetate (F5), methanol (F6), and finally, with water (F7). Each fraction was further tested for in vitro anti-diabetic activities (α-amylase and α-glucosidase) (Figure 5a). Among the seven isolated fractions (F1–F7), the F4 (chloroform) fraction showed the highest inhibition against α-amylase (71.67%) and α-glucosidase (38.01%), followed by F6, F5, F3, F7, F1, and F2.

### 2.6. Determination of Total Phenols and Flavonoids

The total phenolic and flavonoid content of chloroform fraction (F4) of MEUR was estimated by using gallic acid (GA) and quercetin (Q) as standard (Figure 5b,c). The results showed that the total phenolic content (65.19 μg mL^−1^) was higher than the total flavonoid content (20.33 μg mL^−1^).

### 2.7. Purification of Compounds from the Active Fraction (F4)

The active fraction (F4) was further separated using a silica gel column (60–120 mesh) and subjected to thin-layer chromatography (TLC). Among the various solvents, petroleum ether: ethyl acetate in varying ratios yielded five individual compounds. The compounds were purified and characterized using spectrometry using GC-MS, HR-MS, and NMR (^13^C and ^1^H NMR).

#### 2.7.1. Isolation of Compound 1 (Nonane)

**Figure 6:**
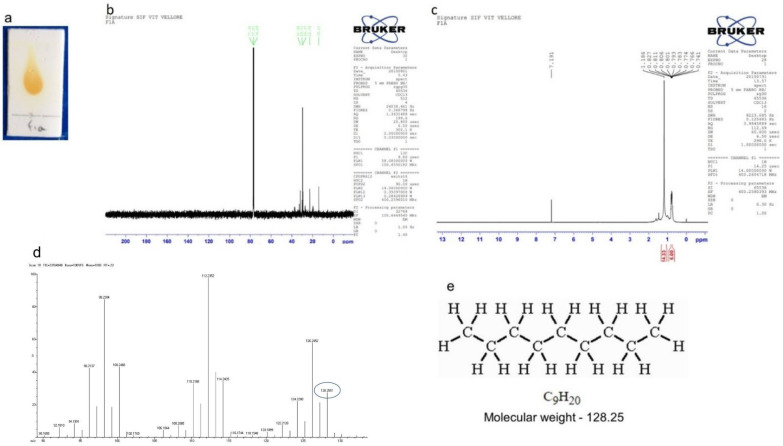
Isolation of compound 1 (Nonane). (**a**) TLC showing a single spot of compound eluted at 100% petroleum ether, (**b**) ^13^C NMR, (**c**) ^1^H NMR, (**d**) HRMS, (**e**) molecular weight and structure.

A colorless liquid compound (39 mg) was eluted from 100% petroleum ether fraction (Figure 6a) and subjected to various spectroscopic analyses for structural determination. The ^1^H NMR (Figure 6b) (CDCl_3_, 400 MHz) δ: 0.74–0.82, (m, 2 × CH_3_, 6H), 1.18 (s, 7 × CH_2_, 14H) ppm; ^13^C NMR (Figure 6c) (CDCl_3_, 100 MHz) δ: 14.1, 22.7, 29.3, 29.7, 31.9 ppm. HRMS (Figure 6d): Calculated for C_9_H_20_: 128.2551; found: 128.2551.

#### 2.7.2. Isolation of Compound 2 (Hexadecanoic Acid)

**Figure 7:**
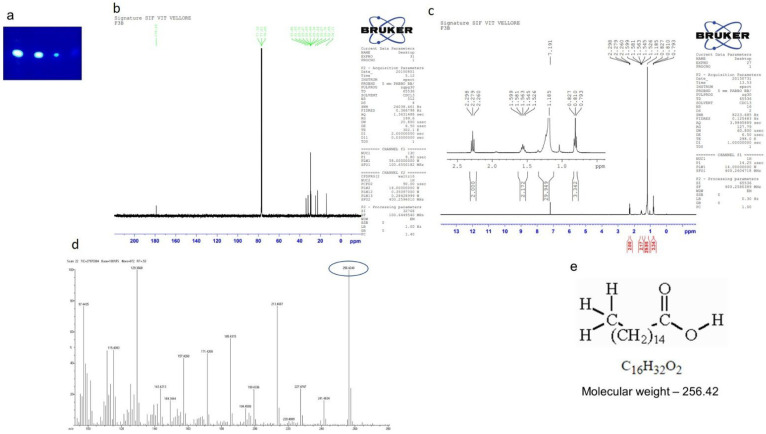
Isolation of compound 2 (Hexadecanoic acid). (**a**) TLC showing a single spot of compound eluted at petroleum ether:ethyl acetate (8:2), (**b**) ^13^C NMR, (**c**) ^1^H NMR, (**d**) HRMS, (**e**) molecular weight and structure.

A white solid compound (75 mg) was eluted from petroleum ether: ethyl acetate (8:2) fraction (Figure 7a). The ^1^H NMR (Figure 7b) (CDCl_3_, 400 MHz) *δ:* 0.79–0.82, (m, CH_3_, 3H), 1.52 (s, 29 H) ppm; ^13^C NMR (Figure 7c) (CDCl_3_, 100 MHz) *δ*: 14.1, 22.6, 24.7, 29.0, 29.2, 29.3, 29.4, 29.5, 29.6, 29.7, 31.9, 33.8, 178.99 ppm. HRMS (Figure 7d): Calculated for C_16_H_32_O_2_: 256.4241; found: 256.4240.

#### 2.7.3. Isolation of Compound 3 (1-dodecanol)

**Figure 8:**
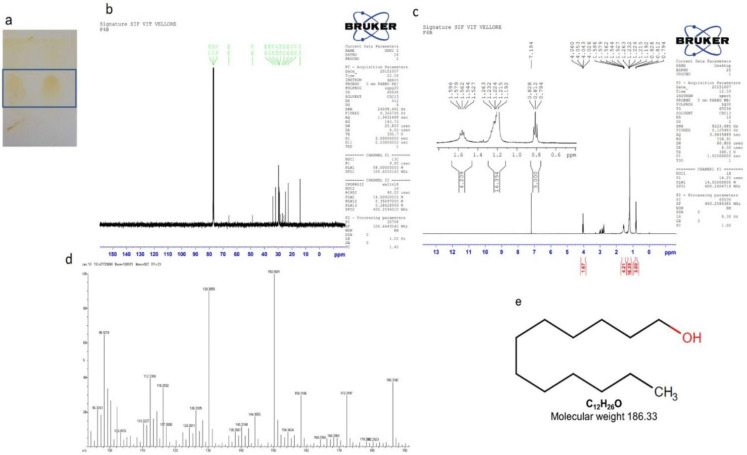
Isolation of compound 3 (1-dodecanol). (**a**) TLC showing a single spot of compound eluted at petroleum ether:ethyl acetate (7:3), (**b**) ^13^C NMR, (**c**) ^1^H NMR, (**d**) HRMS, (**e**) molecular weight and structure.

A pale yellow solid compound (18 mg) was eluted from petroleum ether: ethyl acetate (7:3) fraction (Figure 8a). The ^1^H NMR (Figure 8b) (CDCl_3_, 400 MHz) *δ:* 0.79–0.82, (m, CH_3_, 3H), 1.19–1.26 (m, 17H), 1.52–1.59 (m, 4H), 4.02–4.06 (m, 1H, OH) ppm; ^13^C NMR (Figure 8c) (CDCl_3_, 100 MHz) *δ*: 14.1, 22.7, 24.7, 25.8, 27.0, 29.0, 29.3, 29.7, 31.9, 33.8, 48.7, 65.8 ppm. HRMS (Figure 8d): Calculated for C_12_H_26_O: 186.3342; found: 186.3342.

#### 2.7.4. Isolation of Compounds 4 and 5 (Cyclodecane Methyl and Phenol, Phenol, 3,5-bis(1,1-dimethylethyl))

**Figure 9:**
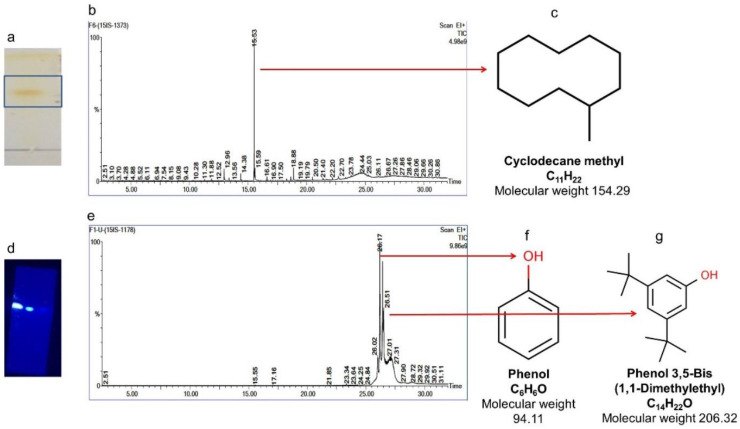
Isolation of compounds 4 and 5. (**a**) TLC showing a single spot of compound eluted at petroleum ether:ethyl acetate (4:6), (**b**) GC-MS chromatogram of cyclodecane methyl, (**c**) molecular weight and structure (**d**) TLC showing a single spot of compound eluted at petroleum ether (100%), (**e**) GC-MS chromatogram of phenol and phenol, 3,5-bis(1,1-dimethylethyl). (**f**,**g**) molecular weight and structure.

The compounds Cyclodecane methyl (15 mg) (petroleum ether: ethyl acetate (4:6)) and a group of phenols (98 mg) (petroleum ether 100%) were eluted in the respective factions (Figure 9a,c). GC-MS analysis confirmed the presence of two compounds and identified them based on the data library available in NIST. The chromatograms obtained are shown in Figure 9b,d.

#### 2.7.5. In Vitro α-Amylase and α-Glucosidase Inhibition Study of Isolated Compounds and Its Active Fraction (F4)

**Figure 10:**
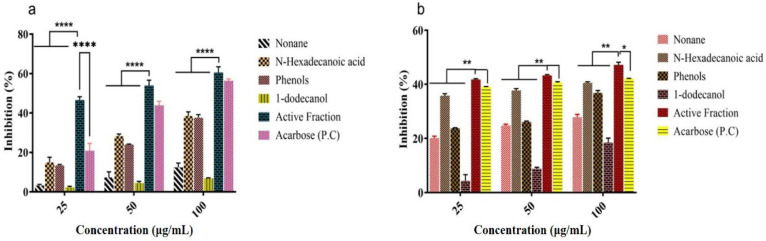
In vitro α-amylase and α-glucosidase inhibitory activity of pure compounds. (**a**) α-amylase and (**b**) α-glucosidase inhibition of isolated compounds and its active fraction (F4). Absorbance was measured at 540 nm and 405 nm. Values are means ± S.E.M (*n* = 3). Mean values were analyzed using two-way ANOVA (Tukey’s multiple comparisons test). **** *p* < 0.0001, ** *p* < 0.01, * *p* < 0.05 were considered significant.

Five different isolated compounds (nonane, hexadecanoic acid, 1-dodecanol, cyclodecane methyl and phenol, phenol, 3,5-bis(1,1-dimethylethyl), and the active fraction (F4) exhibited significant α-amylase and α-glucosidase inhibition [Figure 10a,b]. Among them, the active fraction (F4) showed the highest α-amylase (60.58%) and α-glucosidase (47.18%) inhibition at a concentration of 100 μg mL^−1^. Similarly, n-hexadecanoic acid and phenol, phenol, 3,5-bis(1,1-dimethylethyl) showed α-amylase (38.51 and 37.63%) and α-glucosidase (40.54 and 36.85%) inhibition at a concentration of 100 μg mL^−1^. The results were compared with standard acarbose for α-amylase and α-glucosidase (56.35 and 42.14%), respectively. The IC_50_ value obtained for α-amylase inhibition of active fraction and acarbose was 22.82 and 47.39 μg mL^−1^%, respectively.

### 2.8. In Vitro Insulin Secretion Studies

**Figure 11:**
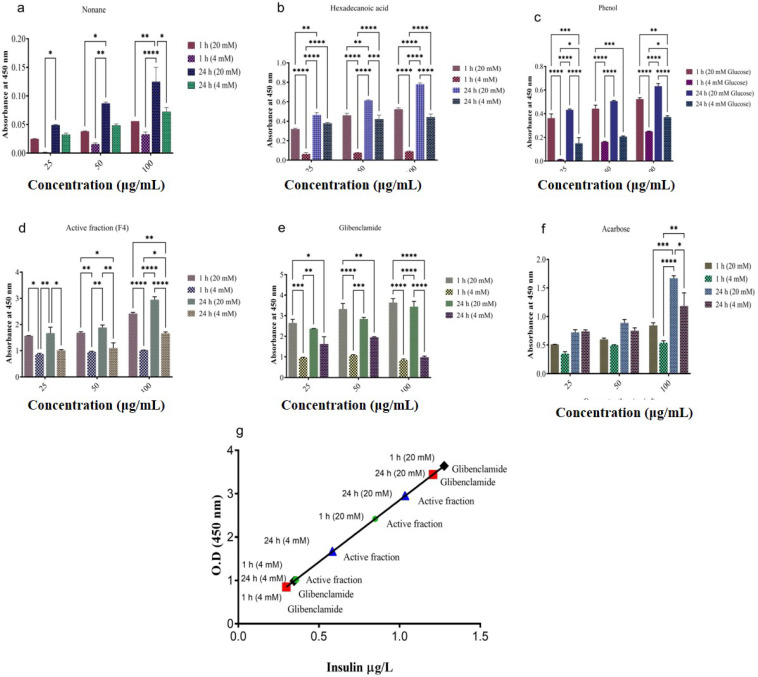
In vitro insulin secretion studies. Effect of isolated compounds and its active fraction (F4) on glucose-induced (4 mM and 20 mM) insulin secretion from pancreatic islets in 1 h and 24 h in vitro culture. Insulin secretagogue activity of isolated compounds and standard drugs (**a**–**f**), the concentration of insulin released in glucose-induced (4 and 20 mM) insulin secretion at 1 and 24 h in vitro culture (**g**). Values are means ± S.E.M (*n* = 3). Mean values were analyzed using two-way ANOVA (Tukey’s multiple comparisons test). **** *p* < 0.0001, *** *p* < 0.001, ** *p* < 0.01, * *p* < 0.05 considered as significant.

The isolated pancreatic islets treated with the test compounds (nonane, hexadecanoic acid, phenol, phenol, 3,5-bis(1,1-dimethylethyl)) and an active fraction (F4) for a period of 1 and 24 h showed significant insulin secretion in both normal (4 mM) and diabetic (20 mM) condition (Figure 11a–d). The two compounds (1-dodecanol, cyclodecane methyl) did not show any effect against pancreatic islets. The results were compared with standard drugs acarbose and glibenclamide (Figure 11e,f). Active fraction (F4) at a concentration of 100 μg mL^−1^ showed maximum glucose-induced insulin secretion at 4 mM (0.357 and 0.582 μg L^−1^) and 20 mM (0.848 and 1.032 μg L^−1^) concentration in both 1 and 24 h in vitro culture. Similarly, glibenclamide (100 μg mL^−1^), a standard antidiabetic drug, also showed a significant increase in glucose-induced insulin secretion at both 4 mM (0.297 and 0.345 μg/L) and 20 mM (1.27 and 1.2 μg L^−1^) glucose concentration in 1 and 24 h time intervals (Figure 11g). Comparatively, acarbose showed less insulin secretion when compared with an active fraction and glibenclamide.

### 2.9. In Vivo Antidiabetic Mechanism of Active Fraction (F4)

**Figure 12:**
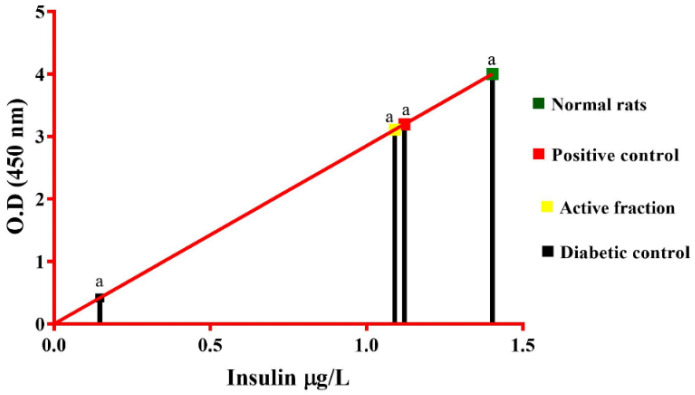
Effect of serum insulin level on experimental rats. The serum insulin level of the experimental groups was measured on the 14th day. ‘a’ denotes serum insulin concentration. Each value is expressed as mean ± S.E.M. (*n* = 6).

Based on the in vitro results obtained from the active fraction (F4) against enzyme inhibition (α-amylase and α-glucosidase) and insulin secretion studies, the fraction F4 was further tested for its hypoglycaemic effect in normoglycaemic animals, glucose-loaded hyperglycemic animals, and STZ-induced diabetic animals. The results obtained from normal, glucose-loaded hyperglycemic, and diabetic rats are shown in Table 3, Table 4 and Table 5. In normoglycaemic, the active fraction (F4) controlled the blood glucose level and showed a similar result as standard acarbose (Table 3). The active fraction (F4) showed a remarkable hypoglycaemic effect at 10 mg/kg B.wt. concentration in glucose-loaded hyperglycemic animals. After 60 min administration of test samples, the blood glucose level was reduced gradually for both active fraction (F4) and acarbose (Table 4). In STZ-induced diabetic rats, the active fraction (F4) showed a 61% reduction in blood glucose level compared to the standard drug glibenclamide (68%). The biochemical parameters of active fraction (F4) were similar to that of the standard drug glibenclamide (Table 5). The serum insulin level of the diabetic control was significantly low when compared to the control. After 14 days of treatment, serum insulin levels of active fraction (1.09 μg L^−1^) and glibenclamide (1.12 μg L^−1^) treated rats were near to normal rats when compared to diabetic rats (Figure 12).

## 3. Discussion

The treatment of diabetes with natural products has been an integral part of traditional medical systems for centuries [31]. In natural resources, phytochemicals with diverse structures belonging to different chemical classes, such as flavonoids, phenols, tannins, alkaloids, terpenoids, steroids, saponins, and polysaccharides are present, which exhibit various bioactive properties [32]. Numerous research findings demonstrate that these natural bioactive compounds can prevent and treat diabetes and obesity by focusing on multiple targets, such as inhibiting carbohydrate-digesting enzymes, targeting activities to improve insulin resistance, insulin secretion, etc. Seaweeds are a potential source of novel compounds exhibiting various bioactivities that could be used in the quest for effective anti-diabetic treatments. An in vitro enzyme inhibitory study was conducted to evaluate the anti-diabetic potential of *Sargassum polycystum* and *Sargassum wightii*, which showed significant inhibitory effects against α-amylase and α-glucosidase and DPP-IV [33]. There were substantial antioxidant activities reported in various seaweed extracts [34,35,36]. Identifying novel antidiabetic compounds with high biomedical value can be accomplished by isolating and characterizing the bioactive compounds and investigating marine algae.

Inhibiting major carbohydrate-digesting enzymes like α-amylase and α-glucosidase and incretin-inhibiting enzymes results in a decrease in glucose absorption rate and, as a result, helps control postprandial hyperglycemia [37]. As part of our previous studies, we have conducted various in vitro and in vivo tests on different marine seaweeds that target this primary mechanism of enzyme inhibition [24,25,33,38,39,40].

In this study, *Ulva reticulata* extracts were investigated for their inhibitory effects on α-amylase, α-glucosidase, and DPP-IV and antioxidant (DPPH) potential using in vitro assays. Among the various extracts tested, the methanolic extract of *U. reticulata* (MEUR) displayed a significant α-amylase (61%), α-glucosidase (97%), and DPP-IV (44%) inhibitory activity among the five extracts. This could be because the seaweed may contain a high concentration of polar bioactive chemicals soluble in highly polar solvents such as methanol. Methanol has been reported to be more efficient in extracting polyphenols with lower molecular weights [41,42]. These findings suggest that methanol is the most effective solvent for extracting bioactive chemicals from *Ulva reticulata*.

*Ulva reticulata* showed moderate free radical scavenging activity. The ethyl acetate extract of *Ulva reticulata* (41%) exhibited the highest free radical scavenging activity, followed by benzene (33%), methanol (30%), and petroleum ether (23%) extracts when compared with the standard BHT (97%).

Seaweed exhibiting potential antidiabetic activity must be evaluated for its efficacy and toxicity to avoid potential dangers such as unwanted side effects, overdose, and toxicity. Measures of cell viability and hemolytic activity of potentially bioactive compounds are essential for developing new drug treatments [43,44]. Furthermore, the measurement of cell viability and in vitro hemolytic activity of MEUR revealed 99% cell viability after 24 h of treatment at 250 μg mL^−1^ and showed no signs of hemolysis or erythrocyte membrane damage. Even at a higher concentration (1000 μg mL^−1^), the MEUR showed significantly less (6.92%) cell lysis, and in the presence of (250 g mL^−1^) MEUR, only 2.81% of human red blood cells were lysed. The extracts have a low to hemolytic impact on human erythrocytes. The obtained results from the study suggest that the MEUR is non-toxic and safe.

Research in animal models is crucial in discovering innovative and effective treatments for chronic diseases like diabetes [45]. The oral administration of MEUR on STZ-induced diabetic rats demonstrated a significant reduction in blood glucose levels (46.21%) compared with glibenclamide (73.05%). Diabetic rats had reduced body weight compared to normal rats, but after administering MEUR and glibenclamide for 28 days, their body weight significantly increased. MEUR- and Glibenclamide-treated rats reduce triglycerides and total cholesterol compared to the untreated, which inhibits hypercholesterolemia and decreases the risk of atherosclerosis [46]. Treated extracts and the standard group also showed a gradual reduction in ALT and AST. This marked the hepatoprotective effect of MEUR. Other parameters like total protein, urea, albumin, and globulin were similar to normal control. In our earlier studies, we have reported the antidiabetic activity of methanolic extract of *Chaetomorpha antennina* in S.T.Z-induced rats model, with results suggesting that at a period of 28 days (250 mg kg^−1^ B.wt) concentration reduced the fasting blood glucose level to 39.97% and that of positive control glibenclamide (0.25 mg kg^−1^ B.wt) was 73.05%, respectively [24].

In our study, MEUR, which exhibited the highest antihyperglycemic activity, was selected for Bioactivity-guided isolation of active compounds responsible for antidiabetic action, and seven fractions were separated (F1–F7). Among the fractions, F4 (chloroform) exhibited considerable α-amylase (71.67%) and α-glucosidase (38.01%) inhibition. Additionally, the fractions were found to be rich in total phenolic (65.19 μg mL^−1^) and total flavonoid content (20.33 μg mL^−1^). The α-amylase and α-glucosidase inhibition is attributed to the presence of phenols and flavonoids. The enzyme inhibition is due to phenols and flavonoids directly binding to enzyme amino acid residues (AARs), preventing substrate binding, or interacting with AARs around the active site, preventing substrate binding [47,48,49].

The active fraction (F4) was further purified by column chromatography and subjected to thin layer chromatography (TLC) using Petroleum ether; ethyl acetate was used in varying ratios as the solvent, and the purified compounds were characterized by employing GC-MS, HR-MS, and NMR (^13^C, ^1^H NMR), techniques. Upon characterization, five distinct compounds—nonane, hexadecanoic acid, 1-dodecanol, cyclodecane methyl, and phenol, 3,5-bis(1,1-dimethylethyl) were identified.

In vitro α-amylase and α-glucosidase inhibition study of isolated compounds and its active fraction (F4) was carried out, revealing active fraction (F4) showed the α-amylase (71.67%) and α-glucosidase (38.01%) inhibition at a concentration of 100 μg mL^−1^ as compared to the individual identified compounds. Carbohydrate-digesting enzymes α-amylase and α-glucosidase are associated with postprandial hyperglycemia; as a result, inhibition of these major enzymes helps reduce glucose release and absorption in the small intestine [50].

Insulin secretagogues lower blood glucose by promoting insulin secretion, boosting insulin levels in the blood, and thereby managing diabetes [51]. Multiple research attempts have been carried out over the last three decades to produce an insulin-secreting beta cell line that maintains normal insulin secretion control, but only a few have been successful [52]. In the present study, non-toxic concentrations of nonane, hexadecanoic acid, phenol, phenol, 3,5-bis(1,1-dimethylethyl), and the active fraction (F4) stimulated concentration-dependent insulin release from isolated mouse pancreatic islets, for a period of 1, and 24 h showed significant insulin secretion in both normal (4 mM) and diabetic (20 mM) condition. Active fraction (F4) at a concentration of 100 μg mL^−1^ showed maximum glucose-induced insulin secretion at 4 mM (0.357 and 0.582 μg L^−1^) and 20 mM (0.848 and 1.032 μg L^−1^) concentration in both 1 and 24 h in vitro culture. The results of the insulin secretagogue activity were compared with standard drugs acarbose and glibenclamide, where maximum glucose-induced insulin secretion was seen in Active fraction (F4) and glibenclamide, and comparatively, acarbose showed less insulin secretion. Moreover, similar results were also previously reported with fucoidan extract of the seaweed *Fucus vesiculosus*, stimulating insulin secretion [51].

The in vivo antidiabetic mechanism of active fraction (F4) was further investigated using various Male albino wistar rat models. The results demonstrated that the active fraction (F4) reduces blood glucose levels in normoglycaemic animals and produces effects similar to that of standard acarbose. Active fraction (F4) demonstrated outstanding hypoglycaemic activity in hyperglycemic animals at a dose of 10 mg/kg B.wt. In the STZ-induced diabetic rat model, the active fraction (F4) showed (61%) reduction in blood glucose level when compared to the standard drug glibenclamide (68%). Significant increase in serum insulin was also observed compared to the diabetic control. Our findings suggest that among the various solvents used, the methanolic extract of *Ulva reticulata* (MEUR) displayed the highest antidiabetic activity in both in vitro and in vivo; it showed no cytotoxicity and hence was subjected to bioassay-guided chromatographic separation. Among the seven isolated fractions (F1 to F7), the F4 (chloroform) fraction exhibited substantial total phenolic content (65.19 μg mL^−1^) and total flavonoid content (20.33 μg mL^−1^), which showed demonstrated the promising inhibition against α-amylase (71.67%) and α-glucosidase (38.01%). Active fraction (F4) was further purified and characterized. Upon structural elucidation, five distinct compounds, namely nonane, hexadecanoic acid, 1-dodecanol, cyclodecane methyl, and phenol, 3,5-bis(1,1-dimethylethyl) were identified. Active fraction (F4) at a concentration of 100 μg mL^−1^ showed maximum glucose-induced insulin secretion at 4 mM (0.357 and 0.582 μg L^−1^) and 20 mM (0.848 and 1.032 μg L^−1^) concentration in both 1 and 24 h in vitro culture, and also exhibited promising antidiabetic activity in various in vivo models. The study’s findings strongly imply that *Ulva reticulata* has the potential to help manage diabetes.

## 4. Materials and Methods

Porcine pancreatic α-amylase, dinitrosalicylic acid (DNSA), acarbose, p-nitrophenyl α-_D_-glucopyranoside, Diprotin A (Ile-Pro-Ile), DPP-IV from porcine kidney, gly-pro-p-nitroanilide (GPPN), streptozotocin, glibenclamide were purchased from Sigma-Aldrich Pvt. Ltd., Bangalore, India. DPPH, butylated hydroxytoluene (BHT), [3-(4, 5-dimethylthiazol-2-yl)-2, 5-diphenyltetrazolium bromide] (MTT) were obtained from HiMedia Laboratories Pvt. Ltd., Maharashtra, India. α-glucosidase and dimethyl sulfoxide (DMSO) were obtained from Sisco Research Laboratories Pvt. Ltd., Maharashtra, India. Silica gel GF 260 plates were purchased from Merck, Darmstadt, Germany. All other chemicals used were of analytical grade, which was available commercially.

### 4.1. Collection of Seaweeds

**Figure 13:**
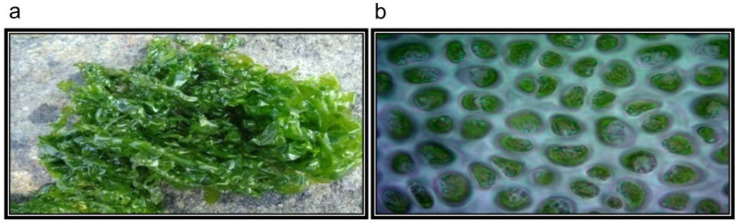
Macroscopic and cross-section image of *U. reticulata.* (**a**) Macroscopic view of *U. reticulata*, which appears like long thalli (mature) and color ranges from light to dark green. (**b**) Microscopic-axial view of *U. reticulata* (magnification 40×).

Fresh seaweed, *U. reticulata*, was collected from intertidal and subtidal regions of Karunagappalli (Latitude 9°3′16″ N; Longitude 76°32′7″ E) Kollam, Kerala (India) in November 2012. It grows attached to rocky substrates, and after it gets mature, thalli easily detach and become free living vegetative algae. Mature thalli have irregular shapes, are light to dark green in color, and range in size from a few centimeters to approximately a meter. (Figure 13). The collected seaweed was identified and authenticated by Dr. P. Kaladharan, Principal Scientist and Scientist In-Charge, Calicut Regional Center of Central Marine Fisheries Research Institute, Kerala (India). A voucher specimen (VMBL-06) was deposited in the Marine Biotechnology and Bioproducts Laboratory, Vellore Institute of Technology.

### 4.2. Preparation of Seaweed Extracts

Collected seaweeds were cleaned, holdfasts were removed, shade dried, and finely powdered. The powdered seaweed (25 g) was extracted with various solvents (250 mL) based on polarity (petroleum ether, benzene, ethyl acetate, acetone, and methanol) using the Soxhlet apparatus for 24 h. Each filtrate was concentrated to dryness under reduced pressure using a rotary evaporator (model-PBU-6, Superfit Continental Pvt. Ltd., Mumbai, India). The samples were lyophilized using a freeze dryer (Penguin Classic Plus, Lark Innovation Fine Technology, Chennai, India) and stored in a refrigerator at 2–8 °C for further use in subsequent experiments.

### 4.3. In Vitro α-Amylase and α-Glucosidase Inhibition Study

The α-amylase and α-glucosidase inhibitory activity of the extracts (250–1000 μg mL^−1^) were determined as described earlier by [40]. Acarbose (250–1000 μg mL^−1^) was used as a positive control. The tests were performed in triplicates, and the inhibitory activity was calculated as percentage inhibition using the formula.

% Inhibition = [(Abs_control_ − Abs*_samples_)/Abs_control_] × 100

### 4.4. In Vitro Dipeptidyl Peptidase-IV (DPP-IV) Inhibition Study

DPP-IV inhibitory activity was determined according to the method by [53]. Seaweed extracts of various concentrations (2.5, 10, 40, and 80 μg mL^−1^) were prepared in Tris-HCl buffer (50 mM, pH 7.5). The assay was performed according to the standardized procedure of Diprotin A (0.2, 0.4, 0.8, 1.6, 3.2, and 6.4 μg mL^−1^) as standard. The tests were performed in triplicates, and the percentage of DPP-IV inhibition was calculated as follows:

% Inhibition = [(Abs_control_ − Abs*_samples_)/Abs_control_] × 100

### 4.5. Free Radical Scavenging Activity (DPPH)

Free radical scavenging activity was determined according to the method of [54]. Seaweed extracts of various concentrations (250–1000 μg mL^−1^) were prepared. Butylated hydroxytoluene (BHT) was used as a positive control. The tests were performed in triplicates. Scavenging activity was expressed as percentage inhibition using the following formula.

% Inhibition = [(Abs_control_ − Abs*_samples_)/Abs_control_] × 100

### 4.6. Maintenance of Cell Line

J774 cell line (mouse macrophage) was obtained from National Center for Cell Science, Pune, India. The cells were grown in Dulbecco’s Modified Eagle’s Medium (DMEM) supplemented with 10% (*v*/*v*) heat-inactivated fetal bovine serum (FBS), 2 mM L-glutamine, and 100 U mL^−1^ of penicillin/streptomycin with 5% CO_2_ at 37 °C in a humidified incubator till passage no. 4. Exponentially growing cells were used for the experiment.

### 4.7. Cytotoxicity Assay

Briefly, cells were seeded at a density of 5 × 10^4^ cells mL^−1^ and allowed to attach for 24 h in 300 μL of medium incubated at 37 °C supplied with 5% CO_2_. After 24 h, various concentrations of seaweed extracts (250–1000 μg mL^−1^) were added to the medium and incubated for 24 h. After incubation, cells were treated with MTT [3-(4, 5-dimethylthiazol-2-yl)-2,5-diphenyltetrazolium bromide] (5 mg mL^−1^) for 4 h. The formazan crystals formed were dissolved in DMSO (Dimethyl sulfoxide) after aspirating the medium. The extent of cytotoxicity was measured spectrophotometrically at 630 nm with a microplate reader (Bio-TEK, Santa Clara, CA, USA).

### 4.8. In Vitro Hemolytic Activity

The hemolytic activity of the crude extracts (250–1000 μg mL^−1^) was evaluated as described by [55].

### 4.9. Experimental Animal

Male albino Wistar rats between 2 and 3 months of age, weighing 180–280 g, were used for the study. Animals were maintained in the animal house, Center for Biomedical Research, VIT, Vellore. Rats were housed in polypropylene cages, maintained under standard temperature conditions (22 ± 2 °C) on a 12 h light and dark cycle. They were fed with a standard rat pellet diet and water ad libitum. Animals were maintained as the principles and guidelines of the Institutional Animal Ethical Committee (IAEC) following the Committee for the Purpose of Control and Supervision of Experiments on Animals (CPCSEA) guidelines on animal care. All animal experiments were approved by the IAEC, VIT/IAEC/10th/14 March/No.26.

### 4.10. Experimental Design

Twenty-four rats were divided into four equal groups (*n* = 6) as follows:

(1) Normal control: Rats fed with normal food and water
(2) Diabetic control: Rats were made diabetic by a single intraperitoneal injection of streptozotocin (STZ) at a concentration of 45 mg kg^−1^ body weight.
(3) Diabetic rats treated with glibenclamide (Positive control): Rats were made diabetic by STZ (45 mg kg^−1^ body weight) and treated orally with standard antidiabetic drug glibenclamide (0.25 mg kg^−1^ body weight) once daily for 28 days.
(4) Diabetic rats treated with methanolic extract of *U. reticulata* (MEUR): Rats were made diabetic by STZ (45 mg kg^−1^ body weight) and treated orally with methanolic extract of *U. reticulata* (250 mg kg^−1^ body weight) once daily for 28 days.

After 3 days of STZ-injection, fasting blood glucose (FBG) values above 250 mg dL^−1^ were considered diabetic. The treatment started on the third day, and diabetic animals were considered for further study and continued for 28 days. FBG levels were measured with a One Touch Select simple^TM^ glucometer, and body weights were checked in regular intervals (0, 7, 14, 21, and 28 days) during the experimental period. At the end of the experiment, the animals were made to fast overnight, and the blood was collected. The collected blood was incubated for 15–30 min at room temperature, the serum was separated by centrifugation (3000 rpm), and the collected serum was used for various biochemical parameters using standard kits (Span Diagnostics Ltd., Surat, India). The animals were sacrificed on the 28th day, and organs, kidneys, liver, and pancreas were collected for histopathological studies.

### 4.11. Bioassay-Guided Fractionation and Isolation of Active Compounds from MEUR

Methanolic extract of *Ulva reticulata* (MEUR), which showed the highest antidiabetic activity in both in vitro and in vivo, was subjected to chromatographic separation using a silica gel column (60–120 mesh). Initially, the powdered seaweed material (200 g) was extracted using Methanol, and the extract was evaporated to dryness under a vacuum using a rotary evaporator (Super Fit-Rotavap, model-PBU-6, India). The methanolic extract (10 g) was further subjected to column chromatography. MEUR loaded onto silica gel column (60–120 mesh) were eluted with various solvents, starting with less polarity solvent in the sequence, such as hexane (F1), benzene (F2), Dichloromethane (F3), chloroform (F4), ethyl acetate (F5), methanol (F6), and finally, with water (F7). Further, each fraction was assayed for in vitro antidiabetic activity (α-amylase and α-glucosidase). Finally, the active fraction (F4) was subjected to TLC, and the individual compounds were separated using column chromatography (60–120 mesh). Later these discrete compounds were identified and characterized based on spectroscopic methods, including NMR (C-13, H-NMR), GC-MS, and HR-MS spectrometry.

### 4.12. Determination of Total Phenols and Flavonoids

The concentration of total phenolic present in the active fraction was determined using Folin–Ciocalteu’s reagent [56]. Briefly, 100 μL of active fraction (F4) of MEUR and 500 μL of Folin–Ciocalteu’s reagent, and 1 mL of Na_2_CO_3_ (20%) were mixed and incubated at room temperature for 90 min. The absorbance was measured at 760 nm. Results were expressed as μg gallic acid equivalents per mg of extract (μg GAE/mg). Similarly, the concentration of total flavonoids in the active fraction was determined according to the aluminum chloride colorimetric method [57]. Briefly, the active fraction (F4) 100 μL was mixed with 95% alcohol, 10% aluminum chloride hexahydrate, 0.1 mL of 1 M potassium acetate, and 2.8 mL of deionized water. After incubation for 40 min at room temperature, the absorbance was measured at 415 nm. Results were expressed as μg quercetin equivalents per mg of extract (μg QE/mg).

### 4.13. In Vitro α-Amylase and α-Glucosidase Inhibition Study of Isolated Compounds and Their Active Fraction (F4)

The α-amylase and α-glucosidase inhibitory activity was determined as described by [58]. Purified compounds and the active fraction (F4) of varying concentrations (25–100 μg/mL) were used for the assays, and acarbose was used as a positive control. The experiments were performed in triplicates, and the inhibitory activity was calculated as percentage inhibition using the formula described previously.

### 4.14. In Vitro Insulin Secretion Studies Using Isolated Pancreatic Islets

Pancreatic islets were isolated from adult male Wistar rats by using the standard collagenase digestion method [59]. Islet cells having viability greater than 90% were chosen for the studies. Insulin secretion study was determined in both 1 and 24 h time intervals to evaluate the effect of isolated compounds and its active fraction against pancreatic islet cells. Therefore, the isolated islets (150 cells/mL medium) were incubated with 5% CO_2_ at 37 °C in a humidified incubator. Test samples of varying concentrations (25–100 μg/mL) were treated with normal (4 mM glucose) and diabetic (20 mM) conditions, respectively. After incubation, cells were centrifuged at 1500× *g* for 15 min at 4 °C. The supernatant obtained was subjected to measure insulin secretion according to the manufacturer’s instruction (Mercodia ultrasensitive rat insulin ELISA, Uppsala, Sweden).

### 4.15. In Vivo Antidiabetic Mechanism of Active Fraction (F4)

#### 4.15.1. Effect in Normoglycemic Animals

Rats fed with normal food and water were made to fast overnight with water ad libitum. The control group received distilled water, and at the same time, positive control (acarbose & glibenclamide) and test samples at a concentration of 10 mg/kg B. wt. were administered using the exact vehicle. FBG levels of each rat were measured at ½, 1, 2, and 4 h after the administration of samples.

#### 4.15.2. Effect in Glucose-Loaded Hyperglycemic Animals

Rats that fasted overnight were administrated glucose (2 g/kg B.wt) after the oral administration of the test and positive control at a concentration of 10 mg/kg B.wt. FBG was measured just before and after the administration of the test samples.

#### 4.15.3. Effect in STZ-Induced Diabetic Animals

Rats were made diabetic by a single intraperitoneal injection of streptozotocin (STZ) at a concentration of 45 mg/kg body weight. After 3 days of STZ injection, fasting blood glucose (FBG) values above 250 mg/dL were considered diabetic. The treatment started on the third day, and animals with diabetes were treated with test samples and the standard antidiabetic drug glibenclamide at a concentration of 10 mg/kg B.wt. FBG levels were measured with a One Touch Select simple^TM^ glucometer, and body weights were checked in regular intervals (0, 7, and 14 days) during the experimental period. At the end of the experiment, the animals fasted overnight, and blood was collected. The collected blood was incubated for 15–30 min at room temperature, the serum was separated by centrifugation, and the collected serum was used for analyzing the insulin content (Mercodia ultrasensitive rat insulin ELISA, Uppsala, Sweden) and various other biochemical parameters using standard kits (Span Diagnostics Ltd., Surat, India).

### 4.16. Statistical Analysis

One-way analysis of variance (ANOVA) and two-way ANOVA followed by Tukey’s multiple comparison tests and Dunn’s multiple comparisons test was used to compare results. Graph-Pad Prism, Version 5, was used for all the statistical analysis. Values were expressed as mean ± SEM, and the level of statistical significance was taken at *p* < 0.05.

## 5. Conclusions

Diabetes is one of the most prevalent pathological conditions affecting healthy living globally and is accompanied by multiple side effects [60]. It is reported that Diabetes mellitus is frequent in both the elderly and the young population [61]. To effectively and efficiently lower postprandial glycemic levels, novel inhibitors with improved preclinical and clinical trial profiles should be found and produced from natural substances. Marine algae (seaweed) can be considered as one of the prospective sources, with highly bioactive unexplored compounds in the ongoing hunt for effective anti-diabetic medicines. Antidiabetic effectiveness profiles for a variety of marine algae have been previously reported [39,40,62,63,64].

Findings from this study indicated that the extract of MEUR (*Ulva reticulata*) and its active fraction F4 examined could be a promising therapeutic agent with better therapeutic efficacy. The extracts and the active fraction exhibited potential anti-diabetic activity with strong inhibitory activity against α-amylase and α-glucosidase, DPP-IV, and antioxidant (DPPH) potential. Active fraction (F4) displayed a prominent in vivo antidiabetic activity due to the presence of five distinct compounds, namely, nonane, hexadecanoic acid, 1-dodecanol, cyclodecane methyl, and phenol, 3,5-bis (1,1-dimethylethyl). The presence of phenols and flavonoids and the isolated compounds in the fractions also validate the antidiabetic action of the active fraction (F4). The in vivo antidiabetic mechanism of active fraction (F4) was investigated, and it was found to have a significant hypoglycemic impact in glucose-loaded, hyperglycemic rats and STZ-induced diabetic animals. Conclusively, our research findings suggest that *Ulva reticulata* and the bioactive chemicals extracted from it are potentially effective and that safe inhibitors of diabetes mellitus can be used to reduce postprandial hyperglycemia.

## Figures and Tables

**Table 1:** Effect of MEUR on fasting blood glucose level. The effect of MEUR on fasting blood glucose levels of STZ-induced diabetic rats. Glibenclamide served as a positive control. Each value is expressed as mean ± S.E.M. (*n* = 6).

Group (*n* = 6)	Experimental Group	Fasting Blood Glucose Level (mg/dL)				
		0th Day	7th Day	14th Day	21st Day	28th Day
I	Normal control	96 ± 3.7	97 ± 4.06	74 ± 1.78	83 ± 3.14	85 ± 4.69
II	Diabetic control	389 ± 20.08	359 ± 25.84	345 ± 12.7	334 ± 21.72	329 ± 14.75
III	Diabetic + glibenclamide (0.25 mg/kg)	349 ± 57.37	267 ± 75.32	197 ± 28.2	131 ± 24.59	87 ± 4.83
IV	Diabetic + MEUR (MEUR extract, 250 mg/kg)	450 ± 12.01	430 ± 23.34	333 ± 20.81	242 ± 31.09	177 ± 27.3

**Table 2:** Effect of MEUR on levels of various serum parameters of STZ-induced diabetic rats. Each value represents mean ± S.E.M (*n* = 6).

Group (*n* = 6)	Experimental Group	Serum Parameters						
		Total Protein (g/dL)	Triglycerides (mg/dL)	Urea (mg/dL)	ALT (IU/L)	AST (IU/L)	Cholesterol (mg/dL)	Albumin (g/dL)
I	Normal control	6.8 ± 0.67	102.4 ± 3.86	8.22 ± 0.2	6.75 ± 0.83	13.43 ± 1.68	54.45 ± 2.24	3.17 ± 0.04
II	Diabetic control	7.04 ± 1.2	221 ± 72.54	17.9 ± 1.8	25.04 ± 3.37	36.42 ± 6.01	82.87 ± 8.5	2.91 ± 0.04
III	Diabetic + Glibenclamide (0.25 mg/kg)	7.15 ± 1.16	128.91 ± 5.64	16.43 ± 2.86	17.38 ± 2.39	14.84 ± 2.73	59.8 ± 8.12	2.82 ± 0.1
IV	Diabetic + MEUR (MEUR extract, 250 mg/kg)	6.14 ± 0.57	120 ± 27.79	16.97 ± 1.28	13.55 ± 1.17	10.01 ± 2.56	65.69 ± 2.24	2.38 ± 0.17

**Table 3:** Hypoglycemic effect of active fraction (F4) in normoglycemic rats.

Parameters	Time Point	Normal Control	Test (Active Fraction)	Acarbose	Glibenclamide
FBG (mg/dL)	30 min	71 ± 5.46	81 ± 5.57	77 ± 2.6	71 ± 1.85
	60 min	72 ± 3.53	64 ± 0.9	68 ± 3.05	66 ± 1.73
	120 min	67 ± 1.45	58 ± 1.52	66 ± 1.32	55 ± 0.87
	240 min	69 ± 7.84	54 ± 1.73	61 ± 0.28	47 ± 2.51

The glucose levels were analyzed at 30, 60, 120, and 240 min in normal rats. Acarbose and glibenclamide served as positive control. Each value is expressed as mean ± S.E.M. (*n* = 6).

**Table 4:** Effect of active fraction (F4) on glucose-loaded hyperglycemic animals.

Parameters	Time Point	Normal Control	Test (Active Fraction)	Acarbose
FBG (mg/dL)	30 min	89 ± 3.21	87 ± 1.45	77 ± 5.36
	60 min	114 ± 2.33	105 ± 3.6	117 ± 4.78
	120 min	122 ± 2.31	77 ± 5.57	72 ± 1.2
	240 min	96 ± 1.73	61 ± 4.67	56 ± 4.37

The glucose levels were analyzed at 30, 60, 120, and 240 min in rats loaded with 2 g/kg B.wt of glucose. Acarbose served as a positive control. Each value is expressed as mean ± S.E.M. (*n* = 6).

**Table 5:** Effect of active fraction (F4) on STZ-induced diabetic animals.

Parameters		Normal Control	Test (Active Fraction)	Glibenclamide	Diabetic Control
FBG (mg/dL)	1st day	95 ± 3.60	257 ± 9.24	301 ± 20.71	292 ± 11.85
	7th day	90 ± 2.08	212 ± 7.00	229 ± 44.08	363 ± 15.6
	14th day	88 ± 1.2	141 ± 14.82	115 ± 7.67	356 ± 19.17
			Net reduction = 61%	Net reduction = 68%	
Total protein (g/dL)		9.22 ± 0.249	9.32 ± 0.05	11.37 ± 0.18	4.54 ± 0.37
Triglycerides (mg/dL)		65.91 ± 7.53	103.81 ± 1.24	117 ± 5.35	215 ± 21.73
Urea (mg/dL)		8.52 ± 0.39	17 ± 2.23	17.76 ± 0.72	21.71 ± 1.63
ALT (IU/L)		7.65 ± 1.55	14.13 ± 2.56	15.9 ± 2.04	24.16 ± 2.79
AST (IU/L)		7.65 ± 1.55	22.36 ± 0.57	17.67 ± 1.01	31.82 ± 3.60
Cholesterol (mg/dL)		59.22 ± 2.95	79.56 ± 0.75	72.63 ± 3.02	99.22 ± 2.83

The effect of active fraction (F4) on fasting blood glucose and serum parameters of STZ-induced diabetic rats. Glibenclamide served as a positive control. Each value is expressed as mean ± S.E.M. (*n* = 6).

## Data Availability

Not applicable.

## Sample Availability

All the samples are available from the authors.

## Abbreviations

MEUR	Methanolic extract of *Ulva reticulata*
S.T.Z.	streptozotocin
PPHG	Postprandial hyperglycemia
B.wt	Body weight
FBG	Fasting Blood Glucose levels
ALT	Alanine transaminase
AST	Aspartate transaminase
